# The Medical Work of the Local Government Board

**Published:** 1893-11-04

**Authors:** 


### The Medical Work of the Local Government Board.

### THE INCIDENCE OF DISEASE AND SANITATION.

This is the case of Kirkheaton. That Kirkheaton stands alone in respect to things sanitary is too much to hope for, and it is selected for notice here, not from any unique characteristics, but because it happened last year to gain for itself the notice of the Central Sanitary Authority. Most unwillingly, it is true; for the Kirkheatonians are always unwilling that their habits and habitations should be investigated by scientific and unsympathetic outsiders. In the first place, it means inquiry, reproof, and other disagreeable things. In the second, and more important, it means the expenditure of money. Kirkheaton and its kindred regard the expenditure of money on sanitation as an extravagance and a folly. Therefore when the County Council of the West Riding of Yorkshire thought it necessary to make an inquiry into the condition of the district, the Local Board of Kirkheaton deprecated this as unnecessary.

It is some excuse that the district is undeniably poor. Of the 691 houses that it contains 550 are rated at less than £5, and 95 per cent. of all the houses are rated under £10. There are only five houses over £20 ratable value. Moreover, it is a district that is falling off in population. In 1881 its population consisted of 2,747 persons living in 681 houses. In 1891 the population had fallen to 2,632, and only 616 houses were occupied. It is evident that to effect any great sanitary improvement in such a place is difficult. Landlords are unwilling to expend any money on houses that may be unlet before long. Tenants of the class who mostly occupy Kirkheaton cannot afford to pay a higher rent for better sanitary conditions, and the public authorities hesitate to undertake any work that necessitates an increase in the rates. The result is a dead-lock, with a tendency to diphtheria. Kirkheaton can hardly improve its own case, and there is none to help it, though it is located in wealthy Yorkshire. Local Government forbids. Kirkheaton can only get what Kirkheaton can afford to pay for. One cannot but ask oneself sometimes if this modern (and mediæval) notion is not being carried too far; if it will not, in the bitter end, make every parish something like the Russian *mir*, with its isolated independence, its lack of consideration for and from its neighbour; if it will not at last reduce Britain to a condition worse than it knew in the days of the Heptarchy, and all national unity be lost in the envy and malice of conflicting parochialisms.

That, however, is a consideration for the statesman, not the sanitarian, whose duty it was simply to point out wherein Kirkheaton was lacking. It was a sufficient task. Two common defects alone were absent. There was no jerry-building, but substantial stone houses, and there was no over-crowding. But some of these solid houses are placed back-to-back, and suffer in consequence from insufficient ventilation. The greatest blots on the district are, however, the sewerage and the means for disposing of refuse. "At Kirkheaton," says Dr. Barry's report, "the greater part of the sewage converges to an old rubble sewer, passes thence into a pipe sewer, from that through a channel in the rock, thence again into a pipe sewer, next flows in a channel on the surface, then again passes into a pipe sewer, and finally discharges into Kirk Ings Beck without any treatment whatever." This method of conveyance leaves ample opportunity for the poisoning of the land, while yet enough foulness will be left to infect the Beck. Nor is the outfall into the stream arranged to be at a part remote from human habitation. It is just 300 yards above a cluster of houses, whose inhabitants complain, and with sufficient reason, of the stench. Kirk Ings Beck must resemble the Rhine as Coleridge describes it in his epigram.

> The river Rhine, it is well known,
> Doth wash the city of Cologne;
> But tell me, nymphs, what power divine
> Can henceforth wash the river Rhine?

As for the arrangements for the disposal of refuse, it might be said, with almost complete accuracy, that they do not exist. A large midden, convenient to the house, and rarely emptied, is the only provision for most families. These middens are sunk below the level of the ground, so that ground-air can filtrate into them and make their contents more offensive. In one instance—at a house occupied by a milk dealer, to increase the danger—there was a large uncovered privy midden containing a quantity of liquid filth, and abutting on the wall of the dwelling.

It says something for the air of the district that in the face of such sanitary arrangements the death-rate averages only 20·25 per 1,000 of the population; but smaller averages than this are to be found even in towns. It is worthy of notice, too, that as the mortality for the last fourteen years averages only 17·7 per 1,000 living, it is evident that the health of the district is not improving. The infant mortality is at the rate of 129 per 1,000 births, which is sufficiently large. It is true that those children who survive the first two years of their existence are strong enough to fight against all insanitary conditions, and have a fair chance of reaching a good old age. Thus of the 58 deaths which took place during 1889, 19 were children under two years of age, and 20 old people over sixty, four of whom had seen their eightieth year. But for this they may certainly thank their constitutions and not the sanitary authorities. The Medical Officer of Health and the Inspector of Nuisances indeed report defects, but it is no easy matter to get them remedied. Of the 139 cases complained of only 64 were attended to, and the defence of the Local Board that those neglected were only of the milder grade of nuisance, has little weight until we know what in their opinion a mild nuisance is. But from one test we may infer at what price they value sanitation. The ten years' expenditure of the Kirkheaton Local Board was £7,839 10s. 2½d., while the ten years' expenditure on nuisance removal reaches the magnificent sum of £2 3s. 10d. And naturally the Local Board object to any interference from the Local Government Board.

If Kirkheaton stood alone it would hardly be worth while to take special notice of its condition. But it is at most a flagrant example of a state of things that exists in many places. The poor and ignorant suffer, sicken, and die for the want of the decencies, not to speak of the conveniences, of life. Medical and other sanitary officers complain till they are weary, and are regarded as themselves the greatest nuisance for their pains. The Local Board is composed of ratepayers who are often unwilling to tax themselves and those who elected them more than they can possibly help. Will not their past economy form their best plea for re-election? And the result is utter neglect of those sanitary precautions of which we talk so much, and on which the health and life of our people depend. The one remedy for this condition of affairs is a strong central power, unbiassed by local meanness, and bent on enlightening local ignorance, which shall force local authorities to do their duty.

